# Behavior of Rats in a Self-Paced Risky Decision-Making Task Based on Definite Probability

**DOI:** 10.3390/brainsci12060795

**Published:** 2022-06-17

**Authors:** Minzhe Yang, Qiangpei Fu, Xu Hu, Baoming Li, Chaolin Ma

**Affiliations:** 1 School of Life Science and Institute of Life Science, Nanchang University, Nanchang 330031, China; minzheyang@email.ncu.edu.cn (M.Y.); fuqp@lglab.ac.cn (Q.F.); hu_xu0101@wuxiapptec.com (X.H.); bmli@hznu.edu.cn (B.L.); 2 School of Basic Medical Sciences and Institute of Brain Science, Hangzhou Normal University, Hangzhou 311121, China

**Keywords:** decision-making, behavior, probability, risk, rat

## Abstract

Risky decision-making (RDM) is when individuals make choices based on the definite cognition for the probabilities of the options. Risk is embodied in the certainty of reward, and the smaller the probability is, the greater the risk will be. As simulated in human behavior paradigms, RDM scenarios in real life are often guided by external cues that inform the likelihood of receiving certain rewards. There are few studies on the neural basis of RDM behavior guided by external cues, which is related to the relative paucity of the animal behavioral paradigms. Here, we established a cue-guided RDM task to detect the behavior of rats making a decision between a small certain reward and a large uncertain reward in a naturalistic manner. The reward of the risk option could be adjusted to observe the change of choice. Our results showed that: (1) rats were able to master the operation of the cue-guided RDM task; (2) many rats were inclined to choose risk rather than the safe option when the reward expectations were equal; (3) rats were able to adjust the decision strategy in time upon a change in risk, suggesting that they have the ability to perceive risk indicated by the external cues.

## 1. Introduction

Risky decision-making is a common psychological process in daily life. When the occurrence of events is unknown, individuals make choices based on the a priori knowable probability of the outcomes [1,2]. Risk is embodied in the certainty of rewards: the smaller the probability is, the greater the risk will be [3]. Risk-taking is an important form of human behavior that has been the subject of numerous social investigations, scholarly analyses and policy debates [4]. Appropriate risk-taking behaviors are beneficial to individual development, but excessive risk-taking may make individuals unable to abide by social order or legal constraints, resulting in serious consequences.

Empirical studies have shown that decision makers do not usually treat probabilities linearly. Instead, they were apt to over weigh small probabilities and under weigh large probabilities [5,6,7]. Individuals differed significantly in their attitudes to take risks, which might be at least partly due to individual characteristics [8]. There is compelling evidence that differences in risk selection have a genetic basis [9,10,11]. The physiological mechanism of different risky decision-making behaviors is still unclear. Many experimental paradigms that utilize decisions involving risk have been used to study human risky decision-making behavior, such as the Cambridge risk task [12], dynamic investment task [13] and choice reversal task [14]. These tasks demonstrate and simulate the decision scenarios faced by individuals in real life through visual cues that provide individuals with explicit information about the likelihoods of obtaining rewards associated with different choices.

Some studies showed that animals could make appropriate choices by evaluating the probability of the reward [15,16]. Modeling risky decision-making behavior in animals could be instructive in studying the neural mechanism of risk perception and delineating the neural substrates of relative pathological behaviors [17,18]. Several tasks have been developed to study the neural basis of risk-based decision-making in rodents, such as the rat gambling task and probability discounting task (PDT) [19,20]. In these tasks, rats associate different actions with choice-outcome contingencies to generate internal representations of each option’s reward, which guides their choice. The internal representation of the risk weight may fluctuate from trial to trial as it is affected by the outcome of each choice. As simulated in human behavior paradigms, risky decision-making scenarios in life are often guided by external cues that inform the likelihood of receiving rewards. Thus, the previous rodent paradigms are suitable for assessing risky decision-making guided by internally-generated information in the absence of explicit cues. It should be noted that some studies have also found the limitations in the previous tasks and initially proposed the new form of a risk-based decision-making task guided by auditory informative cues [21,22].

The psychological process of decision-making behavior includes information integration, effort evaluation and plan construction [23]. Using external cues that represent the information of the external world to guide decision-making is crucial for adaptive behavior, and the deficiency of this ability may affect risky decision-making behavior [24]. The representation of different external cues involves different neural mechanisms. Studies have shown that the manner in which the brain circuitry contributes to guiding choices under cue-generated conditions may differ from those where decisions are guided by internally-generated information [22,25]. However, there are few studies on the neural basis of risky decision-making behavior guided by external cues, which is related to the relative paucity of the animal behavioral paradigms [24].

To fill this gap, we developed a visual cue-guided risky decision-making (RDM) task performed by rats in a self-paced way. In the RDM task, rats make a choice between safe (small/certain) and risky (large/uncertain) options, indicated by different visual cues, to obtain the reward of water. We detected the behavioral performance of rats to clarify their risk bias under the equal reward expectation of risky and safe options, and to investigate the choice strategies of the rats under different risk weights.

## 2. Materials and Methods

### 2.1. Subjects

Twenty-eight male rats (Sprague-Dawley, 8-week-old, 220–280 g) were used in the present study and raised in the SPF feeding room of the Nanchang University Institute of Life Science. The rats were housed in constant temperature and humidity chambers (23 °C, 50–60%) and were subjected to a 12 h light/dark cycle under standard conditions. The rats were randomly grouped into separate cages (450 mm × 290 mm × 255 mm) after weaning (postnatal day 21), and each one of them was handled by the experimenter for five min/day starting three days before training. The animal experiments were carried out following the “Guidelines for the Care and Use of Laboratory Animals” promulgated by Nanchang University.

To motivate behavior, rats were restricted to water 24 h before behavioral training or testing, and they were allowed to drink water freely for 5 min after each training or testing session. Rats had free access to food every day, and to water at least one day per week without participating in training. The body weight of behaviorally-trained rats was monitored to ensure no less than 80–90% of their body weight under free-feeding. Rats were trained in 80 trials in each daily session. During the learning stages of the RDM task, rats were introduced to the next training stage when their operation accuracy reached 80% for 3 consecutive days to avoid overtraining.

### 2.2. Behavioral Apparatus

**Figure 1:**
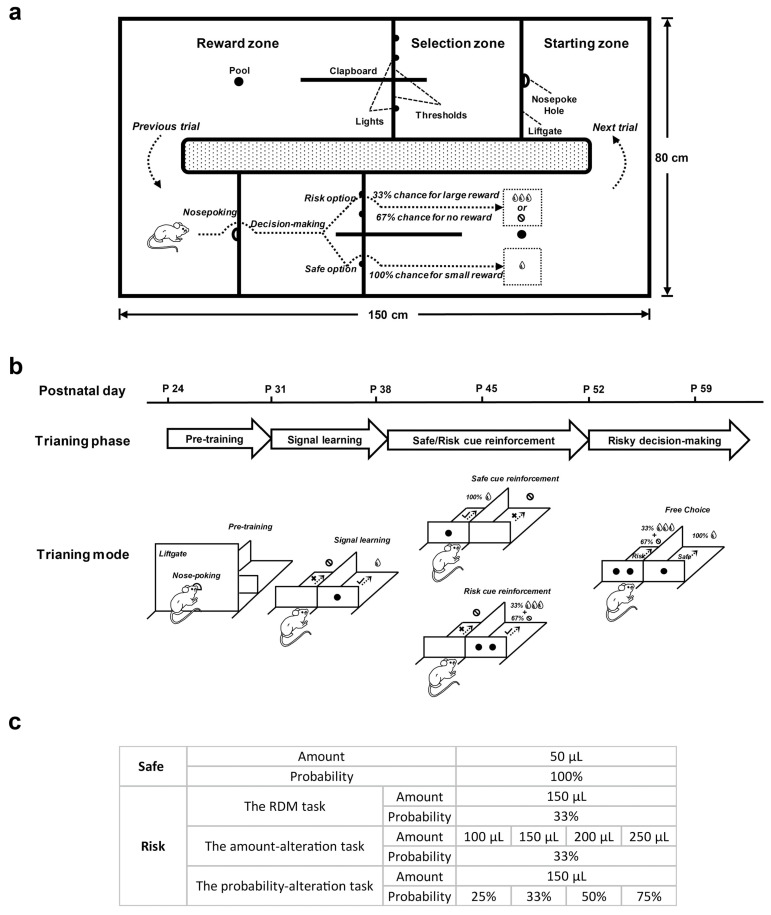
The behavior paradigm for rats to perform the risky decision-making (RDM) task. (**a**) This simplified top view shows the main structure of the RDM maze and the operation of a tested rat in the RDM task. The maze consisted of two identical training chambers placed end to end and therefore formed a closed training loop. The upper part of the diagram shows the key structure of one training chamber, outlined with solid lines and texts. The height of the maze structures was 30 cm except for the thresholds. The lower part of the diagram shows the behavioral operation of a rat in the other training chamber simulated by the dotted arrows and illustrated by the texts. After completing the operation in one training chamber, the rats were required to move counterclockwise to the starting zone of the other training chamber to start the next trial. (**b**) Learning and testing procedures for the RDM task. The learning process included three stages: pre-training, signal-learning and safe/risk cue-reinforcement. The behavior was tested during the risky decision-making stage. The timeline shows the approximate time point at which rats entered the corresponding training or testing stages. The simplified diagram below shows the training or testing modes for each stage. In the pre-training stage, rats were trained to initiate the task by nose-poking and move counterclockwise between the two chambers to perform the task continuously. In the signal-learning stage, rats were trained to understand the significance of LED signal on the threshold in the selection arm. In the safe/risk cue-reinforcement stage, rats were trained to reinforce cues with rewards. In the risky decision-making stage, the choice behavior of rats between safe and risky cues was detected in the free-choice block, which was preceded by two cue-test blocks. (**c**) The reward amount and probability for safe and risky options used in the different versions of the task.

Rats were trained in the RDM maze (150 cm × 80 cm × 30 cm) consisting of two identical training chambers. The two chambers were connected end-to-end to form a training loop, enabling rats to use the two chambers alternately in a counterclockwise manner to execute the task continuously (Figure 1a). Each training chamber had three zones: the starting zone, the selection zone and the reward zone. A liftgate (height: 30 cm) with an aperture mounted into the center for rats to poke their nose was set between the starting zone and the selection zone. The selection zone was set up with two side-by-side channels as the selection arms. Each selection arm had a threshold (height: 5 cm) equipped with LEDs for cue indication. The reward zone was equipped with a drinking pool, where water was pumped by the solenoid valve.

A loudspeaker at the bottom of the maze could output auditory tones (500 ms, 70 dB) to provide feedback on rats’ operation in each trial. When the operation of rats met the requirements in each trial, the loudspeaker played a high-frequency tone (1000 Hz) to remind rats that the operation was correct. In the opposite case, the loudspeaker played a low-frequency tone (300 Hz) to remind rats that the operation was wrong. The maze was set in a sound-attenuating training room and controlled automatically by a personal computer. The performance of rats was monitored by a camera set up above the maze.

### 2.3. Behavioral Paradigm

Rats started the task by poking their nose into the aperture of the liftgate in the starting zone. The liftgate lowered (down to 5 cm) for rats to cross into the selection zone within 5 s after being triggered. The thresholds of the two selection arms randomly displayed cues (one for safe and the other for risk). Rats made a choice within 5 s according to the cue and crossed the selected arm to enter the reward zone immediately. When rats received the reward corresponding to the selected cue at the drinking pool, the liftgate raised and the training chamber temporarily closed. Rats moved counterclockwise to the starting zone of the other training chamber to start the next trial. The two training chambers operated alternately to achieve continuous execution of the RDM task by rats. If rats did not respond within the specified time, the trial ended due to omission and the training chamber was temporarily closed, and the rats had to wait 10 s before the next trial could be initiated.

In the RDM task, the safe cue was represented by one LED light, indicating that rats had a 100% chance of getting 50 μL water (small reward); and the risk cue by two LED lights, indicating a 33% chance of getting 150 μL water (large reward) and 67% possibility of getting nothing. In each trial, the locations (left and right) of two cues were randomly arranged. Rats were rewarded pseudo-randomly when choosing the risk option, i.e., one out of every three risky choices was rewarded. The operation of rats is simulated by a behavioral diagram in Figure 1a. The Video S1 in the Supplementary Materials shows a sample of a rat performing the RDM task.

In the risk-alteration task, the reward amount or probability of risk option was adjusted (Figure 1c), while the safe option remained unchanged (50 μL water with 100% probability). The risk-alteration task included two subversions: amount-alteration task and probability-alteration task. In the amount-alteration task, the reward probability of the risk option was fixed at 33% and the reward amount included four conditions (100 μL, 150 μL, 200 μL and 250 μL water). In the probability-alteration task, the reward amount of risk option was fixed at 150 μL water, and the reward probability had four conditions (25%, 33%, 50% and 75%).

### 2.4. Behavioral Training

The RDM task included three training stages (pre-training, signal-learning and cue-reinforcement) and one testing stage (risky decision-making). The parameters of risk options in the testing stage were consistent with those in the training stages. Figure 1b shows the paradigm of the training procedures of the task.

#### 2.4.1. Pre-Training Stage

Rats were trained to move counterclockwise, operating in the two training chambers alternately, and to initiate the task by nose-poking in this stage. Each rat was placed in the starting zone of one of the training chambers. When the rat poked its nose into the aperture, the liftgate automatically descended. The rat entered the selection zone through the liftgate within 5 s; otherwise, the liftgate was elevated and the rat missed this trial. The rat had to wait 10 s before initiating a new trial if the previous trial was recorded as omission. If the rat entered the selection zone after initiating the task and continued moving to the drinking pool in the reward zone, the solenoid valve pumped out 50 μL water for the rat to drink. Then, the liftgate was elevated and this training chamber turned to rest. The rat moved counterclockwise to the other training chamber for the next trial.

#### 2.4.2. Signal-Learning Stage

Rats were trained to pay attention to the signal displayed by the LED light on the threshold of the selection arm during this stage. When the liftgate descended, one of the two selection arms lit one light on the threshold. In the selection zone, each rat was given 5 s to make a choice and enter the corresponding selection arm. By choosing the arm with the signal on the threshold, the rat could hear a high-frequency tone and receive 50 μL water in the reward zone. If the rat chose the arm without the signal, namely if the rat’s operation was wrong, it would hear a low-frequency tone and receive nothing. If the rat returned after making a choice or did not reach the reward zone within 2 s, it would not be rewarded, and the trial was considered an omission. The rat had to move to the starting zone of the other training chamber and wait for 10 s before the next trial could be initiated.

#### 2.4.3. Cue-Reinforcement Stage

Rats were trained to recognize and reinforce the reward value corresponding to the safe and risk cues in this stage. When the liftgate descended, one of the two selection arms displayed one kind of cue on the threshold. By choosing the arm with the cue on the threshold, the rat could hear a high-frequency tone and receive a reward corresponding to the cue in the reward zone. The treatments of the rats’ wrong operation were the same as in the previous stage. The cue-reinforcement stage was divided into three phases, namely, safe-cue reinforcement, risk-cue reinforcement and random reinforcement for both cues. Each rat first received the reinforcement of the safe cue in daily sessions: it could obtain 50 μL water when the safe arm (the arm with the safe cue) was selected. When the operation accuracy (the percentage of cue-selection trial in a session) reached more than 80% for three consecutive sessions, the reinforcement of risk cue began. The rat had a 33% chance of receiving 150 μL water and a 67% possibility of receiving nothing in the reward zone when choosing the risk arm (the arm with the risk cue). The probability for rats to receive the reward was pseudo-random to ensure correct cognition of the reward probability, that is, one out of every three risky choices was rewarded. After the operation accuracy of the rat reached 80% for three consecutive sessions in the risk-cue reinforcement, the rat received random reinforcement of both cues: the two cues were reinforced randomly in a daily session, alternating among trials.

#### 2.4.4. Risky Decision-Making Stage

Rats were trained to choose freely and tested when the risk and safe cues appeared simultaneously in this stage. The risky decision-making was divided into three blocks, namely the safe-cue test (20 trials), risk-cue test (20 trials) and free-choice (40 trials). The paradigm of the safe-cue test block was the same as in the safe-cue reinforcement, and the risk-cue test block was the same as in the risk-cue reinforcement. Only when the accuracy of cue selection reached more than 80% in the cue-test blocks, could rats be introduced to the free-choice block to measure risk-choice behavior. In the free-choice block, as the liftgate descended, the risk cue appeared in either of the two selection arms and the safe cue appeared in the other. After making the decision, the rat would hear a high-frequency tone and received a water reward in the reward zone corresponding to their choice. The left and right location of the two cues were random in each trial.

### 2.5. Risk-Alteration Tasks

After rats performed the free-choice RDM task stably, they were then introduced to risk-alteration tasks to test their risk perception. In this study, testing for risk perception was performed on 19 rats, with 9 participating in the amount-alteration task and ten in the probability-alteration task. In the amount- or probability-alteration task, the rats were required to reinforce a new reward value of the risk option. The training procedures and the duration of cue-reinforcement were the same as in the RDM task.

### 2.6. Data Acquisition and Analysis

The performance of rats in the maze was recorded automatically by computer. Trials recorded as omission were excluded. The behavior of rats was statistically analyzed in terms of signal selection and location selection, which were expressed as the choice frequency of the cued arm and the preferred location. For each rat, the location (left or right) with a choice frequency greater than 50% during each daily session was considered to be the preferred side. In the free-choice block, the frequency of choosing the risk option was the average over three consecutive days.

Data were analyzed with IBM SPSS Statistics 25 (IBM Corp., Armonk, NY, USA). The data showed in the texts and figures were expressed as Mean ± SEM. For the learning and reinforcement stages, variables across different sessions were tested using a one-way ANOVA with Tukey test for multiple comparisons to determine if performance reached a steady state. For the risk-alteration tasks, the variables across different risk conditions were tested with a one-way ANOVA with Tukey test for post-hoc analyses. An independent sample t-test was used to compare the behavior of rats in the two risk-alteration tasks with the same reward expectation for risk and safe options. The significance criterion was set at *p* < 0.05.

## 3. Results

### 3.1. Rats Were Able to Master the Operation of the Cue-Guided RDM Task

**Figure 2:**
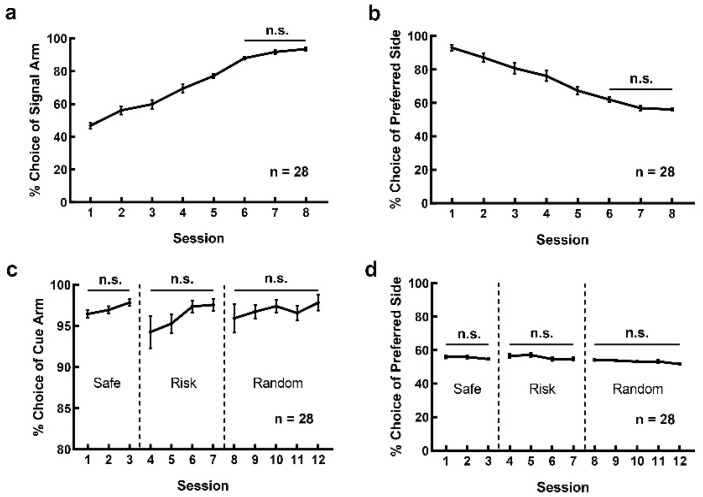
Rats were able to master the operation of the RDM task. (**a**) The learning curve of the rats during the signal-learning stage of the RDM task; (**b**) the location preference, manifested by the uneven location distribution of the selected arm, disappeared with learning sessions during the signal-learning stage of the RDM task; (**c**) performance during the cue-reinforcement stage of the RDM task learning. The safe-reinforcement, risk-reinforcement and random reinforcement phases were separated by the dotted lines. (**d**) There was no location preference for the selected arm during the cue-reinforcement stage of the RDM task learning. (n.s., no significance).

The learning curve in Figure 2a shows that the choice frequency of signal arm gradually increased with learning in the signal-learning stage, reaching over 80% at the 6th daily session and remaining stable in the last three consecutive daily sessions (F(7, 216) = 89.38, *p* < 0.001; Session 6 vs. 7: *p* = 0.835; Session 6 vs. 8: *p* = 0.412; Session 7 vs. 8: *p* = 0.998; other pairs: *p* < 0.05). The location preference gradually decreased and remained stable in the last three consecutive daily sessions (Figure 2b, F(7, 216) = 39.73, *p* < 0.001. Post hoc analysis: Session 6 vs. 7: *p* = 0.712; Session 6 vs. 8: *p* = 0.561; Session 7 vs. 8: *p* > 0.999). The number of trials recorded as omission also decreased with training. From Session 1 through 8, trials with omission were 9.750 ± 0.757, 8.214 ± 0.637, 8.107 ± 0.763, 7.893 ± 0.614, 6.321 ± 0.552, 5.536 ± 0.585, 4.500 ± 0.500 and 4.646 ± 0.526, respectively.

In the cue-reinforcement stage, the performance of rats remained stable (Figure 2c). The frequency of cue selection remained high in each phase of this stage (safe: F(2, 81) = 2.527, *p* = 0.851; risk: F(3, 108) = 1.670, *p* = 0.296; random: F(4, 135) = 0.460, *p* = 0.802). Rats showed no location preference during this stage (safe: F(2, 81) = 0.874, *p* = 0.421; risk: F(3, 108) = 0.787, *p* = 0.504; random: F(4, 135) = 1.981, *p* = 0.101). The numbers of trials recorded as omission in Session 1 through 12 were 3.464 ± 0.387, 2.107 ± 0.339, 2.179 ± 0.334, 7.607 ± 0.543, 6.107 ± 0.528, 4.464 ± 0.323, 2.250 ± 0.270, 2.643 ± 0.322, 1.857 ± 0.250, 1.786 ± 0.288, 2.107 ± 0.201 and 0.929 ± 0.212, respectively.

### 3.2. Many Rats Preferred to Choose Risk Option under Equal Expectation Condition

**Figure 3:**
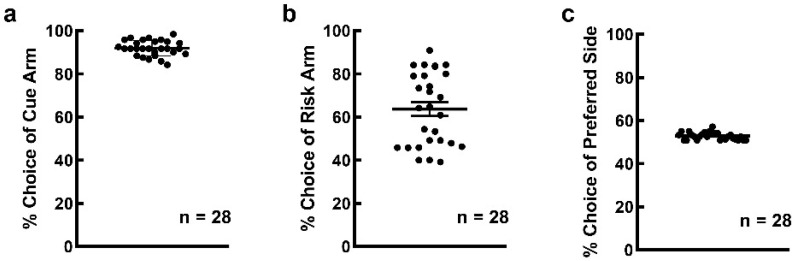
Rats showed preference for risk option in the risky decision-making stage of the RDM task when the reward expectations were equal. (**a**) The mean accuracy was higher than 80% in the cue-test blocks; (**b**) 17 out of 28 rats were inclined to choose risk rather than safe option in the free-choice block, with an average choice frequency of 63.687 ± 3.186%; (**c**) The choice frequency of preferred location was approximately 50% in the free-choice block, indicating that the selection behavior of the rats was independent of the cue location.

In the risky decision-making stage, rats performed well in the cue-test blocks and the average frequency of cue selection was 91.905 ± 0.678% (Figure 3a). The average number of trials recorded as omission was 0.357 ± 0.060 (the cue-test blocks consisted of 40 trials). The rats displayed a marked individual variability in preference for the risk option in the free-choice block. Many rats (17 out of 28) preferred the risk arm, with an average choice frequency of 63.68 ± 3.186% (Figure 3b). Rats had no location preference as the choice frequency of the preferred location was 52.755 ± 0.321% (Figure 3c). Time spent on the free-choice block was 6.071 ± 0.070.

### 3.3. Behavioral Performance Was Modulated by Different Risk Weights

**Figure 4:**
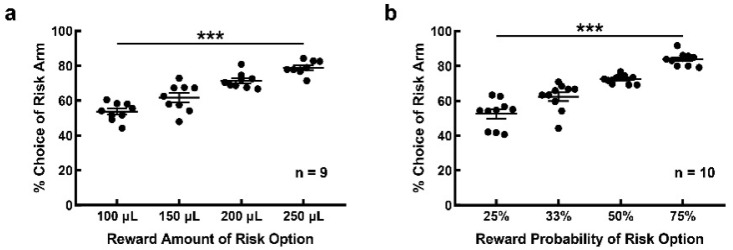
Rats demonstrated the perception of risk weight in the risk-alteration tasks. (**a**) The frequency of choice for risk option increased with the increment of reward amount in the amount-alteration task; (**b**) the frequency of choice for risk option increased with the increment of reward probability in the probability-alteration task. ***, *p* < 0.001.

Nine rats participated in the amount-alteration task. The performance of each rat was an average of its performance over three consecutive days. The risk selection under different amounts of rewards was significantly different (Figure 4a, F(3, 32) = 35.49, *p* < 0.001). When the reward amount for the risk option was 100 μL, 150 μL, 200 μL and 250 μL, the frequencies of the risk choice were 53.694 ± 1.708%, 61.660 ± 2.633%, 71.358 ± 1.459% and 78.848 ± 1.294%. A post hoc analysis revealed a significant difference in risk-choice performance under different reward amounts (100 μL vs. 150 μL, *p* = 0.023; 100 μL vs. 200 μL, *p* < 0.001; 100 μL vs. 250 μL, *p* < 0.001; 150 μL vs. 200 μL, *p* = 0.004; 150 μL vs. 250 μL, *p* < 0.001; 200 μL vs. 250 μL, *p* = 0.035).

Ten rats participated in the probability-alteration task. Risk selection varied with the probability of reward (Figure 4b, F(3, 36) = 47, *p* < 0.001). When the probability of reward for the risk option was 25%, 33%, 50% and 75%, the frequency of risk selection was 52.492 ± 2.625%, 62.292 ± 2.504%, 72.478 ± 0.811% and 83.872 ± 1.168%. A post hoc analysis revealed a significant difference in performance among these reward probabilities (25% vs. 33%, *p* = 0.006; 25% vs. 50%, *p* < 0.001; 25% vs. 75%, *p* < 0.001; 33% vs. 50%, *p* = 0.004; 33% vs. 75%, *p* < 0.001; 50% vs. 75%, *p* = 0.001). No significant difference was detected in the frequency of risk selection between the two groups of rats participating in the amount-alteration task (61.660 ± 2.633%) and the probability-alteration task (62.292 ± 2.504%) under the same reward expectation (risk option: amount × probability = 150 μL × 33%; t = −0.174, *p* = 0.864).

**Figure 5:**
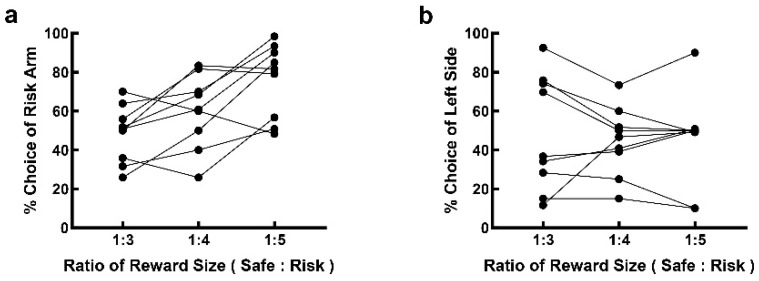
Rats performing poorly in the free-choice block of the RDM task were sensitive to the amount of expected reward in the amount-alteration RDM task. (**a**) Six out of nine rats executed more risk choice with the increase in reward amount for risk option; (**b**) six out of nine rats lost their location preference with the increase in reward amount for risk option.

Several rats (*n* = 9) showed a preference for one location in the free-choice block of the RDM task. We speculated that the same expectation for the two options failed to motivate these rats to make choices. We trained these rats to perform the amount-alteration task. As shown in Figure 5, when the reward amount for the risk option increased to 250 μL, i.e., when the total expectation of the risk option increased by 67%, the choice frequency of the risk option increased, and the location preference disappeared in most of the rats (*n* = 6). Notably, three rats failed to change their preference for location when the risk option was the absolute superior option. This might be the result of a kind of habituation, as the rats became more location-biased with training.

## 4. Discussion

This study provided a detailed behavioral protocol for training and testing rats in a visual cue-guided RDM task. The risky decision-making behavior of the rats was measured in the free-choice block of the RDM task, where rats received 50 μL water when choosing the safe option, and they received 0 μL (67%) or 150 μL (33%) water (the probability was pseudo-random) when choosing the risky option. The safe and risky options were indicated by different visual cues and placed randomly among trials, so that rats could not anticipate the choice behavior before the task was initiated. Our results showed that most rats could be well-trained to perform the RDM task within four weeks from the start of visual signal conditioning. Many rats were inclined to choose the risky rather than safe option when the reward expectations of two options were equal in the free-choice block. The rats were able to adjust their decision strategy in time upon a change in reward of the risk, suggesting that they have the ability to perceive risk.

The maze of the RDM task was adapted from the T-maze [26], and the behavioral paradigm of the RDM task had some similarities to the PDT task. Both tasks required rats to make choice between a smaller, certain reward and a larger reward delivered in a probabilistic manner. Although the two tasks were similar in the setting of choice options, the behavior and cognitive process involved in the tasks were different due to the operating principles. The PDT task required rats to make choices between levers guided by internal representations of reward history, while the RDM task required rats to make choices between paths based on conditioned cues that have been reinforced with reward information. The probability for rats to receive a large reward upon choosing the risky option was pseudo-randomly arranged to avoid the cognitive bias on rare events. The form of cue-guided decision-making in the RDM task drew on the experimental paradigm of human risky decision-making and simulated more common decision-making scenarios in real life.

In terms of the operation methods, the PDT task required rats to make a choice by pressing the lever, while the RDM task required rats to make a choice by entering the selection arm. Rats expended more physical energy in the RDM task due to moving in the maze, which increased the cost of each choice, making it an effort-based RDM task. The RDM task was a naturalistic version of risk-taking in a rat model because rats are more likely to be faced with choices between paths than between levers in the wild. In addition, considering that rats might be induced by the environment or other factors to produce location preference in such a binary choice, the location of the cue in each trial was randomly changed in the RDM task.

In terms of the ways to regulate risk, the PDT task modified the risk level within a session, while the RDM task modified between sessions. The probability in the PDT task increased or decreased by gradient within a session, which was helpful to study the immediate strategy switching in rats but might also produce immediate residual effects on rats. When the risk level was modified between blocks within a session, some rats were unable to accurately perceive changes in risk weight, which could be avoided by changing the risk level between sessions in the RDM task. In addition to the probability of the reward, the amount of the reward also affected the risk weight, which was explored in the RDM task.

From the perspective of reward type, rats were motivated with food to perform the PDT task, while rats were motivated with water in the RDM task. Motivational incentives such as hunger and thirst engaged physiology differently [27,28] and recruited different neuronal circuits [29,30,31], which might affect the risk assessment and the task performance of participants. Studies have shown that water restriction was a more suitable protocol for experiments that required long-term training using a restriction paradigm [32]. Water restriction was less physiologically stressful than food restriction and appeared to promote explorative and cognitive performance in animals while limiting emotional confounds [33]. It has been widely acknowledged that decision-making varied according to individual stress levels [34]. We attempted to reduce the stress level of the RDM task by water restriction to complement and refine the studies of the brain mechanisms of risky decision-making behavior on the existing behavior paradigms.

It was well-known that goal-directed behavior and habitual behavior were two stages of operant conditioning, and we paid more attention to the goal-directed behavior of rats in the RDM task. When training rats to learn or perform the RDM task, habitual behavior could be avoided by the following means: (1) the rats were trained in 80 trials in a daily session and introduced to the next stage in time when their performance reached a stable level; (2) the location of cues in the RDM task was randomly distributed among trials; (3) after the rats made the choice, two tones were used as a feedback signal to indicate whether the operation was performed correctly; (4) the rats with location preference underwent behavior modification or data shaving; (5) cue-test blocks were carried out before the free-choice block; (6) the reward amount or reward probability for risk option was changed to test whether the rats adjusted their decision strategy in time through goal-directed behavior.

Although there are some behavioral paradigms used to study risky decision-making in rodents, the factors influencing decisions were complex and diverse, so it is necessary to develop new behavioral paradigms to explore and clarify the neural mechanisms underlying such complex behavior. Our results based on the RDM task partly replicated the currently available data from previous studies using other tasks. Moreover, some behavioral characteristics of rats in cue-guided risky decision-making were also found.

For convenience, we expressed the reward of the risk option or safe option in the form of reward amount × reward probability in the following statement. Since the reward of the safe option remained fixed (50 μL × 100%) in the amount-alteration task and the probability-alteration task, we normalized the reward expectation of the risk option. In the amount-alteration task, the reward expectation of the risky option was 0.67 (100 μL × 33%), 1 (150 μL × 33%), 1.33 (200 μL × 33%) and 1.67 (25 μL × 33%) times that of the safe option, respectively. In the probability-alteration task, the reward expectation of the risk option was 0.75 (150 μL× 25%), 1 (150 μL × 33%), 1.5 (150 μL × 50%) and 2.25 (150 μL × 75%) times that of the safe option, respectively. The choice of the risk option increased with the increment of the expectation. The results indicated that rats had the ability to perceive the reward expectation of the options.

However, when the reward expectation of the risk option was smaller than that of the safe option, the choice strategy of rats did not conform to the Expected Utility Theory) [35]. That is, when the risk option was inferior (the reward expectation of the risk option was 0.67 times and 0.75 times that of the safe option), the choice frequency of the risk option was higher than that of the safe option (53.694 ± 1.708% and 52.492 ± 2.625%, respectively). The results showed that the reward of the risk option should be large enough to induce rats to pursue, even though the reward was expected to be lower than that of the safe option.

In the amount-alteration task, even if the risky option was inferior to the safe option (the expectation ratio of safety to risk was 1:0.75), the double reward of the risk option was enough to attract the rats to pursue. However, the rats’ preference for the risk option was not unconstrained. When the reward amount of the risk option was 5 times that of safe option, the frequency of the risk choice only increased to 78.848 ± 1.294%. The risk factor of the risk option appeared to be responsible for the ceiling effect in the rat’s choice of risk. Several rats with a preference for selection arm location also participated in the amount-alteration task. The behavior performance suggested that some rats might be more sensitive to the expected values of the reward. When the reward expectation of the two options was the same, the rat’s choice was not related to the cue but to the location of the selection arm, suggesting that these rats’ behavior was habituated based on the location of the selection arm. Rats might not be attracted to make choices as the expected value was equal. When the reward of the risk option increased to the point of absolute superiority, most rats’ choice strategy shifted to a preference for the risk option to maximize the reward.

In the probability-alteration task, even though the reward probability of the risk option was small (25%), the rats chose the risk option slightly more than the safe one (52.492 ± 2.625%). When the risk option was absolutely dominant (the probability increased to 75%), the rats chose the risk option more, but not completely (83.872 ± 1.168%). These results suggested that rats might overestimate the small probability and underestimate the large probability, similar to the nonlinear attitude towards probabilities exhibited by humans. In the case of low reward probability, rats might be attracted by the large reward and therefore chose the more risky option. However, in the case of high reward probability, rats might be more sensitive to risk choices without being rewarded, and the risk aversion led to a ceiling effect in their risk choice.

## 5. Conclusions

Rats were able to master the operation of the visual cue-guided risky decision-making task, and they were inclined to choose risk rather than the safe option when the reward expectations were equal. When the amount or probability of reward for the risk option changed, rats could adjust their choice strategies, indicating that they had the ability to perceive the risk weight. The present study established a more naturalistic and cue-guided risky decision-making paradigm for studying the neural mechanism underlying risky decision-making behavior.

## 6. Patents

The maze of the risky decision-making task is protected by the China National Intellectual Property Administration as Utility Model Patent (patent number: ZL 201720050437.5). The training and testing protocols of the risky decision-making task have been recognized as an invention patent by the China National Intellectual Property Administration (patent number: ZL201710030684.3).

## Data Availability

Not applicable.

## Supplementary Materials

The following supporting information can be downloaded at: https://www.mdpi.com/article/10.3390/brainsci12060795/s1, Video S1: Sample of a rat performing the risky decision-making task.
